# Oropouche Virus: An Overview of the Current Status of Diagnostics

**DOI:** 10.3390/v17101382

**Published:** 2025-10-17

**Authors:** Daniele Lapa, Maria Anele Romeo, Alessandra Spina, Eliana Specchiarello, Fabrizio Maggi

**Affiliations:** 1 Laboratory of Virology and Biosafety Laboratories, National Institute for Infectious Diseases Lazzaro Spallanzani-IRCCS, 00149 Rome, Italy; 2 Department of Bioscience and Agro-Food and Environmental Technology, Universita’ degli Studi di Teramo, 64100 Teramo, Italy; 3 Istituto Zooprofilattico Sperimentale dell’Abruzzo e del Molise ‘Giuseppe Caporale’ (IZSAM), 64100 Teramo, Italy

**Keywords:** Oropouche virus, Oropouche fever, RT-qPCR, serological assay, diagnostics

## Abstract

The *Orthobunyavirus Oropouche* (OROV) has become an urgent public health threat in Central and South America, as well as in other countries worldwide. Since its initial identification, there have been over 30 outbreaks, with the largest reported in late 2024 in Brazil. This outbreak prompted an epidemiological alert due to a significant increase in OF cases in non-Amazonian states in the Americas region, as well as in European countries, where 44 imported cases were identified. Humans become infected predominantly through the bite of the *Culicoides paraensis* midge, and the symptoms could be misinterpreted due to their similarity to those of other arboviral infections. Due to the lack of a point-of-care test, RT-qPCR is currently the key diagnostic test during the acute phase of the disease. This review focuses primarily on the available molecular and serological diagnostic methods. The latter could indeed be used as a confirmation test to monitor the patient’s immunological status and better distinguish between cross-reacting arboviruses. In addition, this review explains also the existing sequencing methods required to enforce the surveillance system for OROV reassortant species that could cause a new worldwide outbreak. The information gathered could provide a valuable basis for implementing additional surveillance systems in those countries lacking up-to-date data.

## 1. Introduction

*Orthobunyavirus oropoucheense* (known as Oropouche Virus—OROV) is an RNA virus with an envelope and 80–120 nm in diameter [1,2]. It belongs to the genus *Orthobunyavirus*, the largest of the five genera of the family *Peribunyaviridae* in the order Elliovirales and causes a febrile illness disease called Oropouche fever (OF). It is part of the Simbu serogroup, which consists of 25 viruses divided into two phylogenetic subclades: Subclade A contains the Manzanilla virus and OROV [3,4], while Subclade B includes Simbu, Akabane, Sathuperi, Shamonda, and Shuni viruses. OROV was first identified and isolated in 1955 in a febrile worker in Vega de Oropouche in Trinidad and Tobago during an outbreak of febrile illness [3,4]. OROV is most likely carried from a forested area into an urban environment by an infected person. Humans become infected predominantly through the bite of the midge *Culicoides paraensis* (Goeldi, 1905) [5] but other insects have also been known to be a potential urban vector. The OROV can also infect people in laboratories. Seven laboratory workers were accidentally infected with the OROV through aerosolisation in the laboratory, and all of them developed symptoms [6]. Recently, a potential sexual transmission of the virus it has been supposed since OROV was also found in the semen of a patient who cleared the infection [7]. This could also lead to speculation about possible sexual transmission, also because OROV could be isolated from seminal fluid up to 16 days after infection.

Since it was first identified, there have been over 30 outbreaks and more than 500,000 cases of OF in Latin America. Recently, the largest OROV outbreak in an extra-Amazonia state in Brazil was reported in late-2024 and early 2025 in the Espirito Santo state with ≥12.000 confirmed cases of OROV by RT-qPCR [8,9]. The number of OROV infections is likely to be underestimated due to misdiagnosis, as the symptoms of OF are similar to those of other viral infections, such as Dengue (DENV), West Nile (WNV), Zika (ZIKV), Mayaro (MAYV), Chikungunya (CHIKV), and Yellow Fever (YFV) viruses [10,11,12].

**Figure 1:**
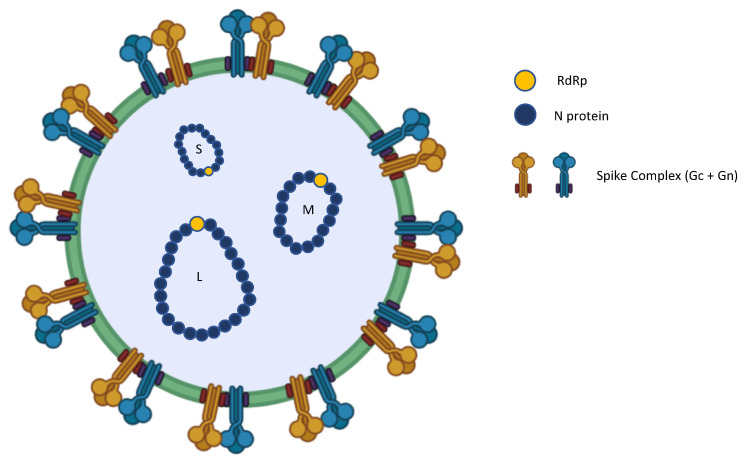
***Orthobunyavirus oropoucheense* (known as Oropouche Virus—OROV) structure.** The OROV genome (-ssRNA) is composed of three RNA segments: small (S), medium (M), and large (L). The S segment encodes the nucleocapsid (N) protein, the medium (M) segment encodes the envelope glycoproteins Gn and Gc, and the L segment encodes the viral RNA-dependent RNA polymerase (RdRp). The image was created in Biorender. Maria Anele Romeo. (2025). https://BioRender.com.

The OROV genome consists of three negative-sense, single-stranded RNA segments: large (L), medium (M) and small (S). Segment L encodes RdRp, which is a critical polymerase for viral genome replication in the cytoplasm of infected cells. Segment M encodes two major antigens (the glycoproteins Gn and Gc), as well as the non-structural protein M (NSM). The S segment encodes both the nucleocapsid protein (N) and the non-structural protein S (NSs) (Figure 1). The S segment usually exhibits the greatest heterogeneity in terms of gene sequencing, and the N protein is indeed the key to classifying OROVs into four genotypes (I, II, III and IV) [13].

**Figure 2:**
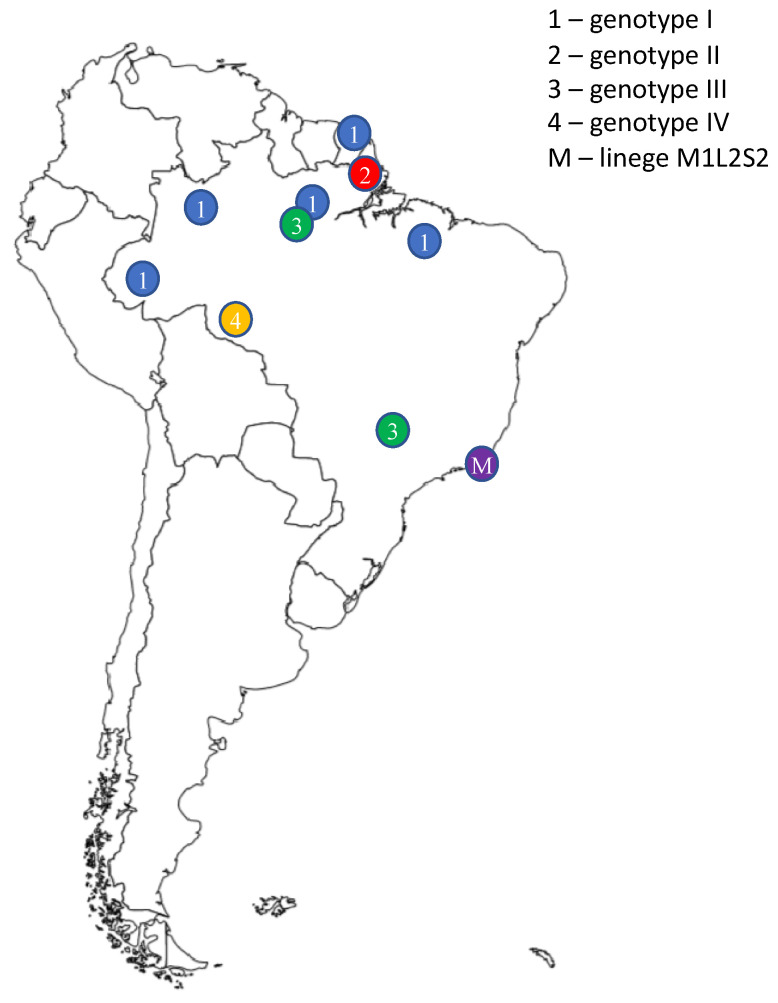
**Distribution of *Orthobunyavirus oropoucheense* (known as Oropouche Virus—OROV) genotypes.** The different genotypes of OROV were first discovered in various regions of South America. The image shows the regions of South America in which they were found. Each number corresponds to a different genotype, as explained in the legend. The image was created in Biorender. Maria Anele Romeo. (2025). https://BioRender.com.

Genotype I was primarily found in the Brazilian states of Maranhão, Acre, Pará and Amazonas, as well as French Guiana. Genotype II is found in Amapá, Brazil. Genotype III is found in Pará, Brazil and Panama, while genotype IV has been identified in Rondônia, Brazil. Additionally, an emergent lineage (lineage M1L2S2) was identified in Rio de Janeiro and was responsible for the Brazilian outbreak in 2024 [14] (Figure 2).

The S segment encodes the N and NSs proteins [15]. The N protein forms the nucleocapsid structure by interacting with the viral RNA and encapsulating and protecting segments of the viral genome. This abundant protein plays a significant role in viral replication and transcription by maintaining the integrity of the viral genome [16].

The NSs protein is critical in counteracting the host antiviral response by inhibiting the host interferon response, thereby facilitating viral replication [17,18].

OROV requires specific cellular components for replication, which takes place in the cytoplasm of infected cells. The viral RdRp, which is encoded by the L segment, synthesises new copies of the viral genome. The assembly and release of OROV are complex processes that are not yet fully understood. It is thought that the synthesised viral RNA and N protein interact in the cytoplasm to form nucleocapsids. These nucleocapsids then migrate to the plasma membrane, where they interact with the Gn and Gc glycoproteins. This leads to new viral particles budding from the cell surface [19].

A study showed that low-density lipoprotein receptor-related protein 1 (Lrp1) supports efficient cellular infection by OROV, although the mechanism is not fully explained. This was demonstrated by observing that OROV infection of Lrp1-deficient cells was significantly decreased [20]. Lrp1 is a ubiquitous, multifunctional receptor involved in endocytosis and signal transduction. This could explain OROV’s ability to infect different cell types [20].

OROV can grow and be isolated in C6/36 and Vero cells, with a strong cytopathic effect becoming evident 5–7 days after inoculation [21,22]. OROV replicates in numerous cell cultures, including BHK-21, MA III, LCM-MK2 and primary chicken embryo fibroblasts. Infection is evidenced by a distinct cytopathic effect, which leads to the destruction of cell monolayers [23]. OROV can also infect other cells, such as liver cells and HeLa cells, as well as astrocytes. During infection of the nervous system, OROV enters neurons via small glial cells, inducing neuronal apoptosis. Although astrocytes are also activated, they do not contribute to neuronal damage [24,25].

Between 2023 and 2025, a significant and rapid increase in OF cases was observed in Brazil, Bolivia, Colombia and Peru. This outbreak had an incidence rate significative higher than the annual median between 2015 and 2023 with a peak in the Espirito Santo state in the early 2025 [8,9]. This significant OF outbreak appears to be associated with new strains of OROV that emerged from complex reassortment events involving multiple S, M, and L segments [11,26,27]. The newly created OROV reassorted strain typically contain the S and L segments from the parental strain, and in most cases, the donor of the M segment is not known [28].

Many studies have demonstrated that OROV reasserted strain are very common. For this reason, a lot of current studies are focused on novel OROV reassortment events that could cause new outbreaks. In a recent study, 21 reassortment events were identified in 133 whole genome sequences, including analysis of the L, M and S segments. These seem to depend on viral adaptation to new environments. Genomic analyses obtained from hundreds of OROV isolates from the most recent outbreak confirm the formation of new genetic reassortments that warrant close monitoring as potential causes of future outbreaks [28]. Recently, it has also been demonstrated that the novel OROV strains arose through complex reassortment events involving diverse S, M and L segments during the Brazilian outbreak of 2024. This led to strains with much higher replicative competence [28]. This fact demonstrated the importance of monitoring of the emergence of novel reassortant strains.

## 2. Immunity

The host immune response to OROV infection is a poorly understood aspect involved in the resolution of the infection. The immune response to OROV infection activates both the innate and adaptive immune systems. Studies have shown that production of interferon and pro-inflammatory cytokines is activated in the early stages of infection [29].

At the onset of viral infection, pattern recognition receptors (PRRs) identify pathogen-associated molecular patterns (PAMPs), initiating a signalling cascade that leads to the production of several cytokines, including interferons (IFNs), which are crucial for containing viral replication. The OROV negative sense RNA genome, a significant PAMP, can be detected by Toll-like receptors (TLRs), which are located on the endosome membrane, or by RIG-like receptors, which are found in the cytoplasm, thus starting this cascade [17,30].

Antigen-presenting cells (APCs) play a vital role in recognising pathogen-associated molecular patterns (PAMPs) and triggering the IFN response via interferon-stimulated genes (ISGs). APCs also contribute significantly to the development of adaptive immunity. One study showed that certain microRNAs, namely miR-217 and miR-576-3p, act as proviral factors during OROV infection facilitating OROV infection by increasing its replication and survival [31,32,33].

The study on necropsy tissue sample of severe case of OROV infection made by multiparametric immunofluorescence identified elevated CD68+ macrophage infiltration in the liver with high levels of pro-inflammatory cytokines IL-6 and IL-17A, suggesting a robust inflammatory cascade contributing to hepatic injury. In contrast, pulmonary tissue revealed IL-4-producing CD68+ cells, suggesting an anti-inflammatory shift that might have failed to mitigate edoema and respiratory compromise [34].

The recent outbreak of OROV in South America has highlighted the urgent need for effective OROV vaccines, which remains a key objective for researchers. Using the live attenuated OROV BeAn19991 strain seems to be a very promising vaccination strategy. Phase I clinical trials showed that this vaccine can induce high levels of neutralising antibodies in vaccinated individuals without causing any serious side effects [35]. Additionally, this strain appears to provide strong cross-protection against different OROV strains. Other strategies include replication-competent vesicular stomatitis virus (VSV) vaccines, subunit vaccines, and immunoinformatics approaches. The latter has enabled several potential T- and B-cell epitopes within the virus to be identified that could stimulate an immune response, offering a promising strategy for vaccine development [2].

## 3. Clinical Aspects

The OF symptomatology lasts for 2–7 days and is associated with fever, chills, intense headache (not sensitive to analgesics), muscle pain, joint pain, malaise, dizziness, nausea, vomiting, sensitivity to light, pain behind the eyes, and, on rare occasions, a skin rash that appears most commonly on the trunk and arms. There may also be haemorrhagic signs, such as spontaneous bleeding, petechiae, nosebleeds, and bleeding gums [7,23,36,37,38]. Physical weakness and loss of strength (asthenia) over a period of 2–4 weeks have been noted in some patients [39]. Central nervous system manifestations such as meningoencephalitis usually occur in immunocompromised individuals and children [40]. Neurological manifestations mainly occur during large outbreaks and include severe headaches, dizziness, lethargy, double vision, involuntary eye movements (nystagmus) and, in some cases, loss of coordination (ataxia), stiffness of the neck (nuchal rigidity), and increased cells in the cerebrospinal fluid (CSF) [37]. The last case of neuroinvasive OROV infection has been described in 2025 in Brazil in an HIV-positive patient. Despite the fact that the death rate was very low in the previous outbreak, the last Brazilian outbreak saw a higher death rate, with nine confirmed deaths and several suspected cases [41].

The incubation period for this disease is three to eight days. Viremia decreases significantly after three days of infection, with reductions in viral load of 72%, 44%, and 23% on days three, four, and five of infection, respectively [42,43,44]. The acute phase of OF commonly lasts 2–7 days and it shows elevated liver enzymes and leukopenia [36,39]. It is possible for symptoms to recur within the first 10 days of the initial symptom onset. This is associated with fever, headache, muscle pain, fatigue, dizziness, and meningitis. The mechanisms responsible for the recurrence of the disease remain unclear [39,42]. In about 60–70% of cases, mild symptoms may recur one to several times 2–3 weeks after their initial manifestation [23,45,46]. This disease affects people of all ages and sexes. OROV should be considered as a differential diagnosis in suspected cases of acute central nervous system (CNS) infection, not only in endemic countries.

The first evidence of pregnancy complications in women with OF emerged during the initial OROV outbreaks in the Brazilian state of Amazonas in 1980. Of the nine infected pregnant women, two miscarried in the second month of pregnancy [47]. Recently, during Brazil’s 2024 outbreak, several cases of pregnant women with OF were reported, in which miscarriage and microcephaly occurred significantly more frequently, confirming the vertical transmission of OROV [48,49,50,51]. It is not known whether OROV symptoms are more severe during pregnancy, as the clinical symptoms of OROV infection in pregnant and non-pregnant women appear to be the same [52,53,54].

## 4. Reservoir Species

**Figure 3:**
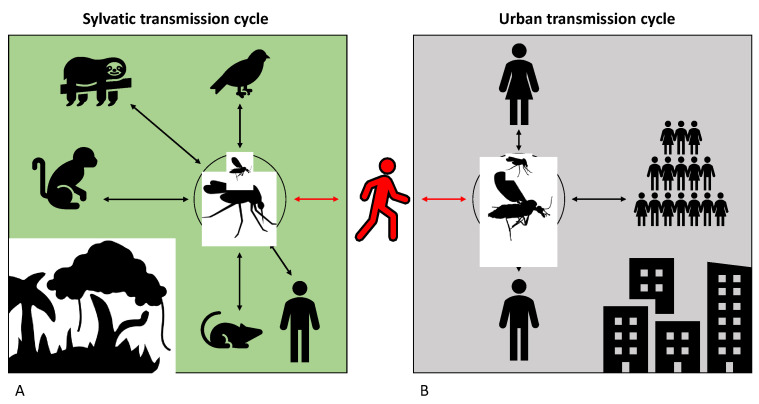
***Orthobunyavirus oropoucheense* (known as Oropouche Virus—OROV) transmission cycle.** The sylvatic transmission cycle (panel (**A**)) and the urban transmission cycle (panel (**B**)) was shown in the image. The persistence of the virus is maintained by the co-occurrence of a sylvatic and an urban transmission cycle in which the midges are the main transmission route. Spillover events may occur when these cycles overlap, either through human intrusion into sylvatic environments or through the movement of sylvatic vectors or animals into urban areas. The image was created in Biorender. Alessandra Spina. (2025). https://BioRender.com.

Many insects within the families Culicidae and Ceratopogonidae have been identified as playing roles in the transmission of OROV, with their involvement varying depending on the geographical context. The persistence of the virus is maintained by the co-occurrence of a sylvatic and an urban transmission cycle, each involving different host species. Wild birds and mammals such as sloths, rodents, and non-human primates are believed to act as reservoirs in the sylvatic cycle, based on serological evidence and sporadic virus isolation in these species [38,55,56,57,58,59]. In contrast, humans appear to be the only vertebrate hosts in the urban cycle. This is supported by studies that have ruled out the involvement of domestic animals, (specifically, cats, dogs, and pigs) based on serological testing conducted during five Brazilian epidemics, which showed no evidence of OROV antibodies [23,39,60]. The biting midge *Culicoides paraensis* has been identified as the primary vector in the urban cycle, while several mosquito species, namely *Coquillettidia venezuelensis*, *Culex quinquefasciatus*, and *Aedes* (*Ochlerotatus*) *serratus* are implicated in maintaining the sylvatic cycle [4,39,43,60,61,62] (Figure 3). Spillover events may occur when these cycles overlap, either through human intrusion into sylvatic environments or through the movement of sylvatic vectors or animals into urban areas [63]. In such cases, humans are suspected to act as bridges between these two cycles, especially since *Culicoides paraensis* is present in both rural and urban settings. Urban outbreaks are often initiated by a viremic individual who was previously infected in a forested area and subsequently returns to an urban environment during the viremic phase [23].

Numerous vector competence studies have been carried out over the years to clarify the identity and capacity of OROV vectors. Experimental infections under laboratory conditions have demonstrated low transmission potential for *Aedes albopictus*, *Culex quinquefasciatus*, *Culex pipiens* and *Anopheles quadrimaculatus* [64]. Another study found that *Culex tarsalis* is probably not a competent OROV vector, whereas the biting midge *Culicoides* (*Monoculicoides*) *sonorensis* exhibited high infection and dissemination potential, although a salivary gland barrier may limit its transmission capacity [65].

Among all studied arthropods, *Culicoides paraensis* is regarded as the primary vector of OROV due to consistent virus isolation during outbreaks and its demonstrated in vivo vector competence under laboratory conditions [37,39,47,55,56,60,66,67]. It belongs to the genus *Culicoides* (Order: Diptera, Family: Ceratopogonidae), which comprises about 1400 species distributed globally, except in New Zealand and polar regions [68]. Approximately 96% of *Culicoides* species are hematophagous, with females requiring blood meals for egg production, and feeding on both humans and other mammals. This behaviour makes them important public health threats due to their ability to transmit arboviruses of human and veterinary significance [13,43,68,69]. While the full host range of *Culicoides* midges remains uncertain, available data suggest they are primarily mammalophilic and/or ornithophilic, feeding opportunistically on mammals and birds depending on host availability [69,70]. The life cycle of *Culicoides paraensis* includes an egg stage, four larval instars, a pupal stage, and an adult (imago) stage, with a life span of 10–20 days [71]. These midges breed in moist environments suitable for egg survival and hatching, such as soil-water interfaces or highly organic substrates including fruit wastes like bananas, cacao husks, and plantains, facilitating human-midge contact in peri-urban areas [62,69,72]. *Culicoides paraensis* exhibits a wide geographic distribution due to its adaptability to semi-urban environments near human populations. Its abundance increases during hot or rainy periods [73,74,75,76]. Unlike most *Culicoides* species, which are crepuscular, *Culicoides paraensis* is active during the daytime [69,72,77].

This biting midge exhibits a neotropical distribution, ranging from the southern United States to Argentina [78,79]. Notably, *Culicoides paraensis* was detected in Cuba for the first time in 2024 [80], corresponding to the OROV outbreaks reported that same year [81]. This species had not been previously captured in Cuba using standard trapping methods (e.g., BG-Sentinel, New Jersey traps, or CDC light traps), but was successfully captured through human landing catches, possibly explaining its previous absence from surveillance reports [80]. These findings highlight the potential for unidentified areas of vector presence and underscore the need for enhanced entomological surveillance.

## 5. Epidemiology

OROV was first isolated in 1955 from a forest worker with a fever in Trinidad and Tobago near the Oropouche River and, subsequently, identified during an epidemic in Brazil in 1961, where it is currently endemic. Indeed, since the first identification, over 30 OF outbreaks have been reported in different regions of Brazil, in particular in the Amazonas region, with the major outbreaks reported in 1980–1981, 2006, 2007–2008 and 2011–2012 [82]. Additionally, it has been demonstrated that not all cases of OROV infection in the Amazon state were diagnosed. A study evaluating the seroprevalence of OROV in at least 20 Amazonian municipalities from 2011 to 2016 found 3% positivity in serum samples negative for DENV, ZIKV and CHIKV [83].

Probably caused by the spreading across the riverbanks of the Amazonas River, facilitated by human mobilisation, in 1992 OROV was detected also in Perú during a serosurvey for a DENV outbreak [23]. Following its initial identification, the virus became endemic in this territory, with several subsequent outbreaks being identified. In addition, OROV circulation was identified in other South American countries, including Bolivia, Ecuador, Colombia and Venezuela, during epidemiological surveillance studies. The broad distribution of the primary vector, rising global temperatures and increased rainfall, as well as urbanisation, created favourable conditions for vector proliferation and virus transmission. As a direct consequence, OROV demonstrated significant geographic expansion and high potential to infect new hosts in the 2000s [84]. Although most reported cases continue to occur in the Brazilian and Peruvian Amazon, an outbreak was known to occur in Colombia between 2019 and 2022 [85]. However, environmental and ecological factors caused OROV outbreaks in French Guiana in 2020 [86] and Haiti in 2014 [87].

In 2024, various cases of OROV fever were reported across South America and the Caribbean. In addition, the Pan American Health Organization (PAHO) issued an epidemiological alert due to a significant rise in OROV disease cases in Brazil, Colombia, and Peru [88]. During the 2024 outbreak, autochthonous transmission was identified in ten non-Amazonian states [89]. Following these outbreaks, 44 cases of OROV disease were reported in EU countries (imported cases), primarily in Spain, Italy, Germany and France, from June to July 2024. Of these, 43 were linked to the outbreak in Cuba and one to the outbreak in Brazil [90,91].

Between epidemiological weeks 1 and 4 in 2025, 3765 confirmed OF cases were reported in the Americas Region [92]. The 2025 outbreak also led to some imported cases in Europe in the first half of the year among travellers who had visited Brazil and Dominica [90].

## 6. Diagnosis

Clinical diagnosis of OF is challenging due to its similar pathogenesis to that of other endemic viral infections that cause febrile illness, such as ZIKV, YFV and DENV [84]. Additionally, the absence of commercial kits complicates the laboratory diagnosis of OROV, indeed the development of reliable in-house techniques is dependent on specialised laboratories.

**Figure 4:**
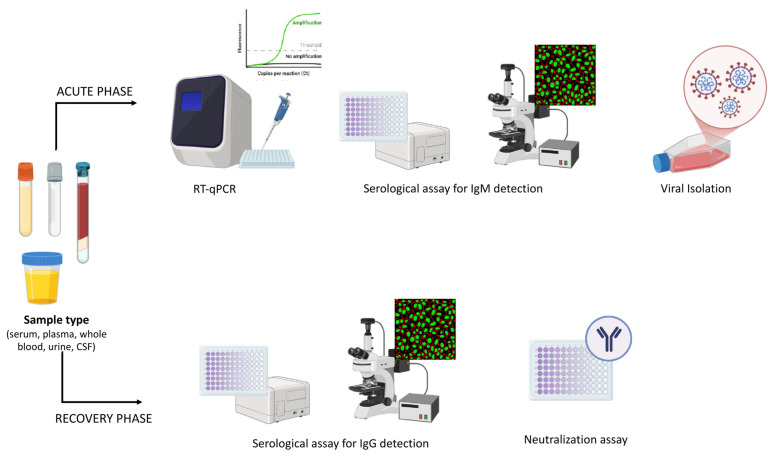
***Orthobunyavirus oropoucheense* (known as Oropouche Virus—OROV) diagnostic approach.** The figure shows the most commonly used diagnostic approaches. During the acute (symptomatic) phase, RT-qPCR is the key diagnostic test. Samples that can be analysed using RT-qPCR include serum, plasma, whole blood, urine and cerebrospinal fluid (CSF). Serological assays for the detection of IgM anti-OROV, which can be performed on serum, plasma or CSF, are only supportive of the diagnosis in this phase and must be confirmed by analysing seroconversion to IgG during the recovery phase. Additionally, a neutralisation assay can be used to evaluate the presence of neutralising antibodies during the recovery phase. The image was created in Biorender. Maria Anele Romeo. (2025). https://BioRender.com.

OF can manifest differently in different patients. The acute phase usually begins 3–8 days after the bite of an infected vector and lasts 2–7 days. Approximately 60% of clinical cases relapse within 10 days, except in cases involving the central nervous system (CNS) [93]. As shown in Figure 4, according to Pérez-Restrepo et al. [80], the first step in making a rapid diagnosis during the acute phase of the disease is to immediately check for viral expression by RT-qPCR (the key test for diagnosis). In addition, serological tests searching for the IgM antibody was used as additional test to evaluate OROV antibodies 4–5 days after the onset of symptoms. In this phase, virus isolation and titration can also be performed, but this requires 7–10 days of work by highly specialised personnel in a BLS-3 laboratory. At the end of the acute phase (one or two weeks after the symptoms onset), it is essential to perform a serological test to evaluate the conversion of IgM antibodies to IgG antibodies, in order to validate the previous serological test. The persistence of the IgG antibody could be evaluated months after the onset of symptoms to monitor the long-term immunity of the patients. The neutralisation capability of IgG antibodies can also be evaluated using neutralisation assays. Different studies have demonstrated the persistent detection of OROV by RT-qPCR in urine and whole blood samples up to 22 days after the onset of symptoms, whereas OROV was only detectable in serum and plasma samples during the acute phase of the disease [7,94]. The persistent detection of the virus in whole blood may be favoured by the presence of a virological reservoir consisting of human T-cells and monocytes for OROV [44,95]. Persistent viremia was associated with strong IgM and IgG positivity (IgM 1:40 and IgG 1:2560, measured at 12 weeks) [7].

An adequate and rapid diagnosis, using the correct samples, followed by reporting new cases to public health authorities, could be useful for the early detection of travel-associated cases or to eventually control an outbreak [95]. With this aim, we would like to give an overview of the diagnostic tests currently available.

### 6.1. Molecular Diagnosis

#### RT-qPCR

Currently, the molecular diagnosis of OROV primarily relies on RT-qPCR. This technique enables highly specific and sensitive detection of the virus, allowing for its differentiation from other arboviruses with overlapping clinical presentations, such as DENV and CHIKV. The preferred clinical specimen for OROV detection is serum, although recent studies have demonstrated that saliva and urine are also viable alternative matrices [96,97]. Most RT-qPCR assays target the S segment of the viral genome, a region known for its high sequence conservation [22].

One of the earliest and most influential studies was conducted by Weidmann et al., who developed a panel of RT-qPCR assays for the detection of various Orthobunyaviruses, including OROV. Their assay demonstrated a limit of detection (LoD) of 100 copies/reaction, identifying 28 out of 30 positive samples (93.3%), compared to only 8 out of 30 (26.6%) detected by the reference nested PCR [98,99].

Although this assay remains valid, more sensitive methods have since been developed to detect not only OROV but also reassortant species derived from it, such as Madre de Dios virus (MDDV), Iquitos virus (IQTV), Perdões virus (PERDV). To this end, a highly conserved region (≥95%) at the 5′ end of the S segment was identified as a suitable target for a degenerate primer/probe-based RT-qPCR assay. The 95% lower limit of detection (LLOD) was determined to be 5.6 copies/µL for H75 and 10.8 copies/µL for BeH [100].

For differential diagnosis, Naveca et al. developed a duplex RT-qPCR assay capable of simultaneously detecting MAYV and OROV. The assay demonstrated high sensitivity (LoD: 2 copies/reaction for MAYV, 20 copies/reaction for OROV), amplification efficiency >98% and high specificity, confirmed against a panel of 17 arboviruses with no cross-reactivity. This assay is particularly valuable in regions where multiple arboviruses co-circulate and has been recommended by PAHO as a diagnostic tool for a differential diagnosis [101,102].

To identify natural reassortment events among OROV strains, Nunes et al. developed novel assays based on primer targeting the M segment, which is more prone to selective pressure than the S and L segments and thus useful for phylogenetic discrimination. The authors performed different PCR formats demonstrated that RT-qPCR have a highest sensitivity (LoD: 0.59 copies/µL) compared to the end-point PCR (LoD: 5.9 × 10^2^ copies/µL) and the nested PCR (LoD: 5.9 copies/µL) [86].

With the increasing incidence and geographic spread of OROV, more advanced diagnostic technologies have been developed. For instance, Pomari et al. adapted the Naveca RT-qPCR protocol into a droplet digital PCR (ddPCR) assay for precise detection and quantification of OROV RNA [103]. Silva et al. introduced a reverse transcription loop-mediated isothermal amplification (RT-LAMP) assay as a rapid and cost-effective alternative [104]. All the in-house PCR assays described above are summarised in Table 1.

Although RUO (Research Use Only) kits for OROV RNA detection are commercially available (Table 2), further validation is required before clinical implementation.

### 6.2. Isolation

To confirm the diagnosis of OROV, the virus can be isolated from serum in Vero E6 or C6/36 cell culture. This technique takes 7–10 days, during which time highly specialised personnel must evaluate the cytopathic effect (CPE) in BSL-3 laboratories.

OROV replication takes place in the cytoplasm of infected cells, where synthesised viral RNA and the N protein interact to form nucleocapsids. These nucleocapsids then migrate to the plasma membrane, where they interact with the Gn and Gc glycoproteins to form new viral particles [19]. According to the STAR protocol [80], a 5–6 × 10^6^ cell culture of Vero E6 or C6/36 can be exposed to various dilutions of the patient’s serum (e.g., 1:10 or 1:100) for one hour. The infected culture medium is then removed and replaced with a medium supplemented with 2% FBS. Specialised personnel then monitor the CPE and recover the supernatant containing the virus when the CPE reaches approximately 70%. Evaluation of the CPE is critical because an infection that is too advanced causes a rapid decrease in viral titres.

### 6.3. Serological Diagnosis

Serological techniques are not actually the key test for OROV diagnosis. Serological assays can indeed be used to aid diagnosis 4–5 days after the onset of symptoms [79,110]. As for molecular methods, sample types that can be used for a serological assay include serum, plasma or CSF in cases of suspected neurological disease. OROV antibodies of the IgM type are usually detectable in serum on the fifth day after the onset of symptoms, but a diagnosis based on a single serum sample in the acute phase of the disease is presumptive. Therefore, it is essential to collect a second serum sample one or two weeks after the initial evaluation to confirm the infection by evaluating either the seroconversion of antibodies (IgM to IgG) or a four-fold increase in antibody titres [111]. The most widely used serological assay is the Enzyme-Linked Immunosorbent Assay (ELISA), which enables the detection of OROV-specific IgM and IgG antibodies, using as antigen recombinant nucleocapsid protein or other viral proteins, allowing successful differentiation from other arboviruses [100]. In addition, a strength of this assay is that it does not require a biosafety level 3 (BSL-3) laboratory, although only a few commercial kits are currently available. Therefore, this technique requires the development of an in-house approach using recombinant antigens [91]. Other serological analyses that allow antibody responses to be evaluated include indirect immunofluorescence, which is based on infected cell lines. This requires an in-house approach to be developed in BLS-3 laboratories, which could complicate the diagnostic tool. In addition, convalescent sera, plasma and/or CSF can be used to perform a complement fixation test (CF) and/or a haemagglutination test (HI) [5], which can detect the presence of OROV-specific antibodies in patients’ samples. Lastly, as with other arboviral infections, a neutralisation assay, such as the plaque reduction neutralisation test (PRNT), is recommended more than seven days after the onset of symptoms [79], with particular attention paid to specimens collected from pregnant women [51]. The PRNT determines the level of neutralising antibodies against OROV by utilising the cytolytic activity of viruses that form plaques in cell cultures. This test measures neutralisation capability based on the number of plaques in the presence or absence of the patient’s serum [80]. Although the PRNT has a longer turnaround time than other common serological methods and requires a BLS-3 laboratory and specialised personnel, it is the most specific serological test and can be helpful in distinguishing between cross-reacting arboviruses [53].

### 6.4. Viral Sequencing

Advancements in sequencing technologies have enabled the acquisition of partial and complete genomic sequences from various OROV strains, significantly enhancing our understanding of the virus’s evolution and geographic dissemination. While the S segment remains the most extensively studied, increased focus on the M and L segments has led to the identification of multiple reassortment events [96,97,98,112,113,114]. Vasconcelos et al. (2011) conducted the first comprehensive study aimed at elucidating the molecular epidemiology of this human pathogen [98]. Phylogenetic analysis of samples collected between 1961 and 2009 resulted in 66 complete N gene sequences and 36 partial sequences of the M and L segments, revealing the existence of at least four distinct genotypes (I–IV), with an average nucleotide divergence of 4.6% in the N gene. Genotype I was identified as the most ancient and widespread, whereas genotype IV was exclusively detected in Brazil, specifically in the state of Rondônia. Importantly, this analysis was conducted using only partial sequences of each gene, rather than complete genome sequences.

Subsequent phylogenetic studies focusing on the N gene have consistently confirmed the presence of four distinct OROV genotypes, as reported in multiple investigations [45,87,96,97,98,114,115].

In 2019, Nunes et al. performed full-genome sequencing of 35 viral isolates using Next Generation Sequencing (NGS) based on random amplification [100]. Their findings corroborated the established genotypic classification and led to the development and validation of novel molecular assays (RT-qPCR, RT-nested PCR) with high sensitivity and specificity. These diagnostic tools are critical for enhancing epidemiological surveillance and response capabilities in endemic regions.

A subsequent study employing a combination of NGS, Sanger sequencing, and Rapid Amplification of cDNA Ends (RACE), along with reanalysis of sequences available in GenBank, proposed a revised classification into four clades (A–D), which better reflects the observed genetic diversity [17]. In their analysis of 10 viral isolates, Tilston-Lunel et al. identified four distinct reassortment events among OROV strains, including a highly divergent M segment in two isolates from marmosets (Callithrix penicillata), leading to the proposal of a novel virus, termed Perdões virus. All the sequencing approach are summarised in Table 3.

In the context of OROV’s expanding geographic range, the study by Deiana et al. (2024) represents a pivotal contribution to the genomic surveillance of emerging pathogens [111]. The authors reported the complete characterisation of the first OROV strain isolated in Europe, obtained from a patient returning from Cuba. Identification and sequencing were performed using an untargeted metagenomic approach on the Illumina NextSeq1000 platform. This strategy enabled the detection of genomic reassortment events and the identification of a novel phylogenetic cluster (OROV_SCDC_2024), underscoring the virus’s ongoing evolutionary dynamics.

The phylogenetics analyses carried out in samples derived from Brazialian Amazon outbreak in 2024, instead, demonstrated that all the samples belong to the clade lineage OROVBR-2015-2024, which has been responsible for the ongoing outbreaks since 2015 [116].

The availability of complete OROV genomic sequences provides a critical reference for the development of diagnostic tools, molecular monitoring, and the elucidation of viral adaptation mechanisms in non-endemic settings.

## 7. Discussion and Conclusions

According to the World Health Organization, OROV is an emerging arbovirus that requires particular attention worldwide [110]. Climate change, which leads to an increased presence of the reservoir species worldwide, misdiagnosis due to similar pathogenesis to that of other arboviral infections, and potential sexual transmission, could cause new outbreaks. The higher death rate measured in the last outbreaks [34], the confirmed ability to be vertically transmitted with an high capacity to cause miscarriage and microcephaly [49], and the virus’ neuroinvasive ability poses a strong concern for the future outbreaks. Rapid diagnosis and monitoring of new reassortant species could help to control the circulation of OROV [18,111]. The key test for OROV diagnosis remains RT-qPCR during the acute phase of the disease. This could target the S segment, which is more conserved across species [100], or the M segment to better detect reassortant species [96]. Continuing to validate the new in-house approach could help to find a common, rapid technique that could be used worldwide. Viral sequencing techniques are also fundamental to monitoring reassortment events and conducting phylogenetic analyses [96,97,98]. The use of these methods aids immediate case recognition and enables health authorities to accurately characterise the distribution of OROV infections and implement preventive control measures.

To date, there is no validated point-of-care diagnostic tool. Rapid point-of-care tests can be used in the laboratory under emergency conditions and would provide an alternative that does not require handling of clinical specimens and extraction of nucleic acids from samples so that an initial diagnosis can be given quickly. These rapid tests should be easy to use but compatible with biosafety and do not require well-trained personnel, making diagnosis faster. Lastly, future investigations are essential to increase our knowledge of the pathogenesis of OROV in order to develop effective vaccines and antiviral treatments.

## Figures and Tables

**Table 1:** In-house molecular assays for OROV detection.

Type of Test	Target Gene	Type of Sample	Performance Parameter	References
RT-LAMP	S segment	OROV strain	LoD: 24.54 copies/reaction	[102]
RT-qPCR	S segment	synthetic sequences, viral strains and serum samples	LoD: 5.6 copies/µL (H75) and 10.8 copies/µL (BeH)	[99]
RT-nested PCR	M segment	OROV isolates	LoD: 5.9 copies/mL	[96]
RT-qPCR	M segment	OROV isolates	LoD: 0.59 copies/mL	[96]
Multiplex RT-qPCR	S segment	cell supernatants and non-human samples	LoD: 2–20 copies/reaction	[100]
RT-qPCR	S segment	serum samples	100 RNA molecules	[99]
Nested PCR	S segment	serum samples	NA	[24]
ddPCR	S segment	whole blood, serum, urine	LoD: 1 copies/mL	[103]

**Table 2:** Commercially available molecular assays for OROV detection.

Test Name	Manufacturer	Test Type	RUO/IVD	LoD	Type of Samples	Internal Control	References
Oropouche Virus Real-Time PCR Kit	Creative Diagnostics (Shirley, NY, USA)	Real-Time PCR	RUO	LoD: 2.5 copies/reaction	serum	NA	[105]
Oropouche Virus Nucleic Acid Detection Kit	Creative Biogene (Shirley, NY, USA)	Real-Time PCR	RUO	NA	serum, plasma	Yes	[106]
Oropouche-Mayaro Virus Real-Time PCR Kit	Vircell (Granada, Spain)	RT PCR Multiplex	RUO	NA	serum, plasma, whole blood	Yes	[107]
RealStar^®^ Oropouche Fever RT-qPCR Kit 1.0 RUO	Altona Diagnostics (Milan, Italy)	Real-Time PCR	RUO	NA	serum, plasma, whole blood	Yes	[108]
Oropouche Virus Real-Time PCR Kit (RUO)	Bioperfectus (Taizhou City, China)	Real-Time PCR	RUO	2.5 copies/reaction	serum	NA	[109]

**Table 3:** Sequencing approach for OROV detection.

Type of Test	Target Gene	Type of Sample	Performance Parameter	References
Sanger sequencing + seminested PCR	S, M and L segments	plasma, saliva and urine	NA	[18]
NGS by random amplification	S, M and L segments	OROV isolates	NA	[96]
NGS and Sanger sequencing	S, M and L segments	clinical isolates and non-human samples	NA	[11]
Sanger sequencing	S, M and L segments	OROV isolates	NA	[98]

## Data Availability

No new data were created or analysed in this study. Data sharing is not applicable to this article.

## Abbreviations

The following abbreviations are used in this manuscript:

APCs	Antigen-presenting cells
CF	Complement fixation test
CNS	Central nervous system
CSF	Cerebrospinal fluid
ddPCR	Droplet digital PCR
ELISA	Enzyme-Linked Immunosorbent Assay
HI	Haemagglutination test
IFNs	Interferons
IQTV	Iquitos virus
ISGs	Interferon-stimulated genes
L	Large
LLOD	Lower limit of detection
LoD	Limit of detection
Lrp1	Lipoprotein receptor-related protein 1
M	Medium
MAYV	Mayaro virus
MDDV	Madre de Dios virus
N	Nucleocapsid protein
NGS	Next Generation Sequencing
NSM	Non-structural protein M
NSs	Non-structural protein S
NT	neutralisation test
OF	Oropouche fever
OROV	Oropouche virus
PAHO	Pan American Health Organization
PAMPs	Pathogen-associated molecular patterns
PERDV	Perdoes virus
PRRs	pattern recognition receptors
RACE	Rapid Amplification of cDNA Ends
S	Small
TLRs	Toll-like receptors
WHO	World Health Organization
